# Urinary tract fungal ball in a single kidney after total nephroureterocystectomy

**DOI:** 10.1002/iju5.12757

**Published:** 2024-07-09

**Authors:** Yoshinori Nakano, Yohei Sekino, Kenshiro Takemoto, Takeshi Ueno, Shogo Fujii, Hiroyuki Kitano, Keisuke Hieda, Shinya Ohara, Nobuyuki Hinata

**Affiliations:** ^1^ Department of Urology Nakatsu Daiichi Hospital Ooita Japan; ^2^ Department of Urology, Graduate School of Biomedical and Health Sciences Hiroshima University Hiroshima Japan

**Keywords:** Candiduria, fungal ball, nephroureterocystectomy, single kidney, urinary tract

## Abstract

**Introduction:**

Fungal balls in the urinary tract are rare but dangerous complications of candiduria. Here, we report a case of a urinary tract fungal ball in a single kidney after total nephroureterocystectomy.

**Case presentation:**

The patient was an 80‐year‐old male. He had a history of pyelonephritis, and his ureteral stent was regularly replaced. He was admitted to the hospital with a chief complaint of urinary tract obstruction, and a 50‐mm‐sized mass was found within the renal pelvis. Because the ureteral stent frequently became obstructed, the mass was removed percutaneously. *Candida albicans* was detected on the tissue culture results, and the mass was diagnosed as a fungal ball.

**Conclusion:**

Appropriate urinary drainage methods for fungal balls vary among patients, and it is important to select an appropriate method based on the accumulated number of cases.

**Keynote message**

We experienced a urinary tract obstruction caused by a 50‐mm‐sized mass within the renal pelvis in a single kidney. The mass was completely removed percutaneously and diagnosed as a fungal ball. To the best of our knowledge, this is the first reported case of a urinary tract fungal ball in a single kidney after total nephroureterocystectomy.

**Abbreviations & Acronyms**
CT: computed tomography
HPF: high‐power field

## Introduction

Symptomatic urinary tract fungal infections require therapeutic intervention and are often caused by Candida species. In rare cases, fungal balls form in the urinary tract. This is a dangerous complication that makes treatment difficult. To the best of our knowledge, there have been no reports of urinary tract fungal balls occurring in a single kidney. Here, we report a case of a urinary tract fungal ball that developed after emphysematous pyelonephritis.

## Case presentation

The patient was an 80‐year‐old male who underwent a total left ureterocystectomy and right ureterocutaneous fistula surgery for bladder cancer. The patient underwent outpatient treatment for bladder cancer without recurrence and was receiving oral diabetes mellitus treatment. The patient had a history of pyelonephritis due to stricturing of the ureterocutaneous fistula. At that time, a ureteral stent was placed, and *Proteus mirabilis* was detected in urine culture. Ceftriaxone were administered for 2 weeks. After which the ureteral stent was regularly replaced. He occasionally experienced fever and was treated for pyelonephritis with antibiotics.

**Fig. 1:**
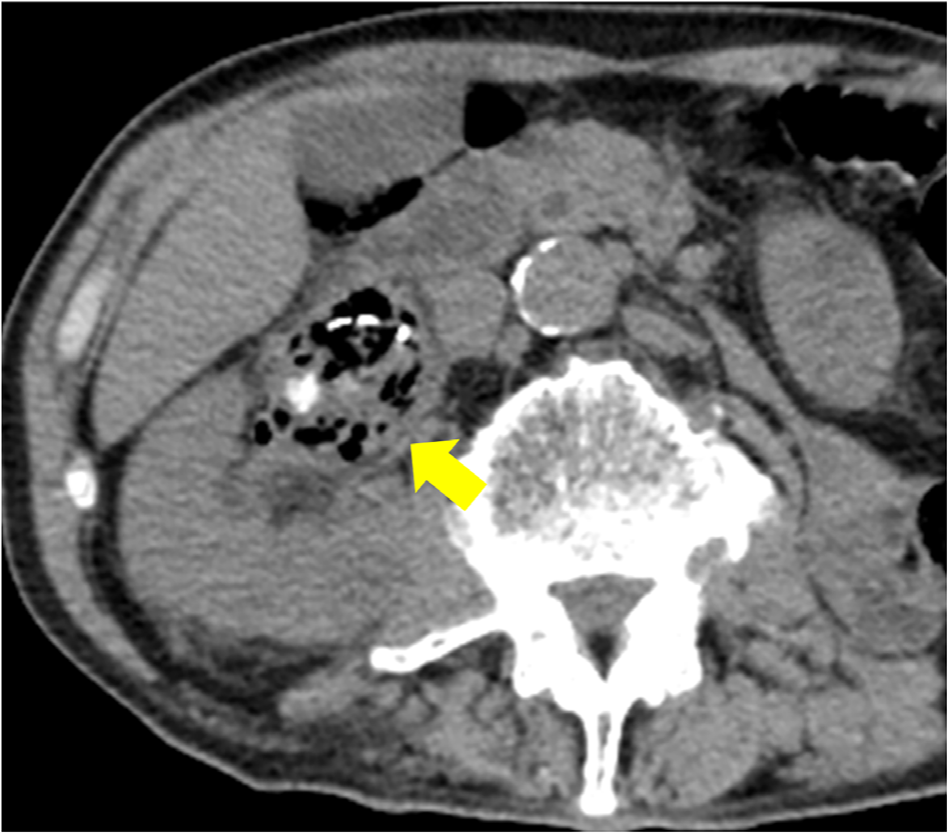
A mass that was separate with the renal pelvic wall, with air and calcification inside was, found within the renal pelvis. Because of the simple CT, it was difficult to assess the continuity between the renal pelvis wall and the mass.

**Fig. 2:**
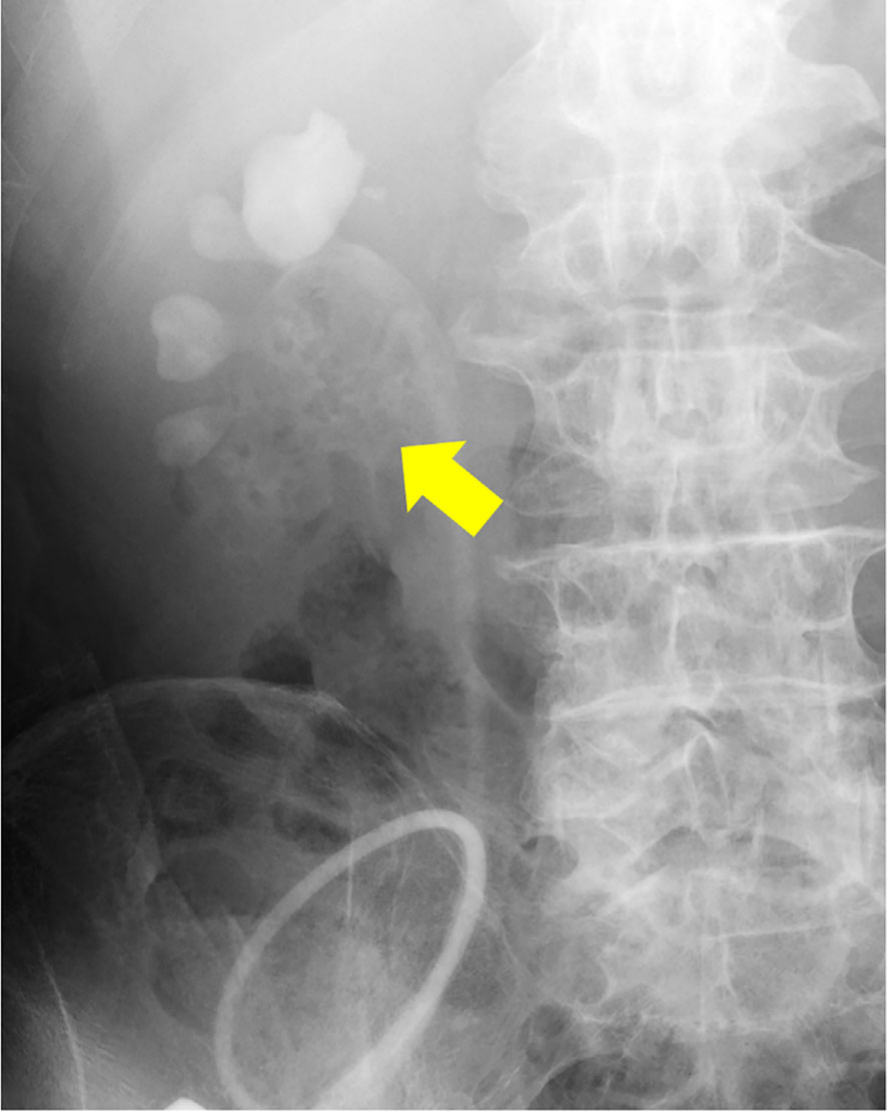
A shadowed defect measuring 64 × 39 mm is seen within the renal pelvis. The interior of the shadows is uneven and appears to have no continuity with the renal pelvic wall.

In August 2022, he visited our hospital with the chief complaint of urinary tract obstruction. The patient did not have a fever or right lower back pain. When the stent was replaced, it was obstructed by yellowish‐white debris. Laboratory investigations revealed a total leukocyte count of 14.7 × 10^9^/L and a serum creatinine level of 2.55 mg/dL. His original serum creatinine level was 1.51 mg/dL. His blood sugar levels were properly controlled, and no hyperglycemia was observed. Urinalysis revealed microhematuria (97.2 red blood cells/HPF) and leukocyturia (1187 white blood cells/HPF). Computed tomography (CT) revealed a mass that was contained air (Fig. 1). Retrograde urography revealed a shadowed defect measuring 64 × 39 mm (Fig. 2).

**Fig. 3:**
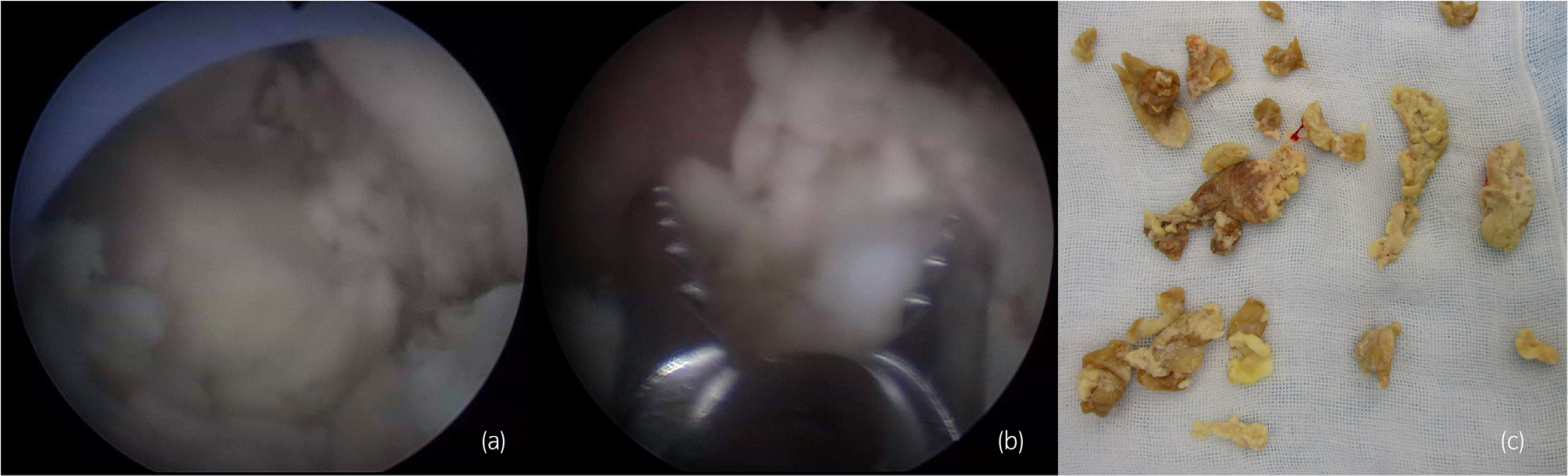
Surgical findings. A nephrostomy was added, and the mass was removed using monitoring through a pyeloscope. Large yellowish‐white soft mass is visible within the renal pelvis under pyeloscope (a). The fungal ball was removed using grasping forceps (b). By using a thick outer tube and sufficient reflux, even relatively large debris could be extracted, allowing for efficient surgery. Extracted fungal ball (c).

Antibiotic therapy was initiated with meropenem, *Proteus mirabilis*, and *Candida albicans* was detected in urine cultures. Observation under a flexible ureteroscope revealed a large, yellowish‐white soft mass within the renal pelvis. At that time, an attempt was made to remove the tissue using a basket catheter, but the tissue could not be removed effectively. Because the patient's condition did not improve, we decided to perform percutaneous removal. A nephrostomy was performed, dilated with a dilator, and a 30Fr outer tube was inserted into the renal pelvis. A pyeloscope was inserted, and the mass was completely removed using forceps (Fig. 3). *C. albicans* was detected in the tissue culture results, and the tissue was diagnosed as a fungal ball.

After surgery, there was no urinary tract obstruction, and we decided to manage with nephrostomy, the nephrostomy catheter was replaced regularly. Although no additional treatment was given, no fungal infections have recurred to date.

## Discussion

Upper urinary tract fungal infections are relatively uncommon, and fungal ball formation is particularly rare.^1^, ^2^ Fungal balls can originate from the agglutination of necrotic tissue nuclei, mucosal debris, and foreign or lithiasic debris. This can lead to urinary tract obstruction and hydronephrosis and may become quite large.^3^ In cases of a single kidney, it may cause postrenal renal failure. The pathogens most frequently observed to cause fungal balls were *Candida albicans* and *Candida tropicalis*.^4^ Diabetes mellitus, urinary tract abnormalities, urinary catheters, prolonged antibiotic therapy, steroids, immunosuppressive therapy, and malnutrition are well‐known risk factors for urinary tract fungal infections.^4^ There are two mechanisms of pathogenesis of urinary tract fungal diseases: urinary tract infiltration associated with fungemia and retrograde urinary tract fungal infections.^5^ Although the direct relationship between a history of emphysematous pyelonephritis and fungal balls is unknown, in this case, we thought that the relatively long‐term use of antibiotics after the onset of emphysematous pyelonephritis may have become a risk. It is also thought that the patient's diabetes mellitus may have contributed to the fungal infection. However, because blood cultures could not be evaluated, the possibility of fungemia cannot be ruled out.

CT typically shows fungal ball as a filling defect in the urinary tract.^6^ Contrast‐enhanced CT to evaluate the continuity with the renal pelvis wall can differentiate a mass from a tumor^7^, but contrast‐enhanced CT scan was not performed because of mildly elevated renal parameters in this case.

The treatment of urinary tract fungal infections includes the administration of antifungal drugs and drainage. In cases of fungal balls, a combination of antifungal drugs and drainage has often been reported.^4^, ^8^, ^9^ The most commonly used antifungal drugs are fluconazole and amphotericin B deoxycholate (AmpB) because the urinary concentrations of other drugs are very low.^10^ However, it is necessary to adjust the dosage of antifungal drugs for patients with renal dysfunction.^11^ There are several drainage methods. It has been reported that when the fungal ball is small, surgery can be performed transurethrally, and the fungal ball can be removed using a basket catheter.^12^ However, if the fungal ball is large, it is difficult to separate it into smaller pieces because of its viscosity. Ultrasonic lithotripsy used to treat stones are ineffective on fungal balls.^13^ The advantage of percutaneous surgery is that using a large‐caliber working channel allows for easy fragmentation/removal/suctioning of the entire fungus ball.^6^ If the fungal ball is difficult to remove, nephrectomy may be considered depending on the risk.^7^ Additionally, injecting antifungal drugs into the renal pelvis through a nephrostomy tube and sucking with negative pressure have been reported as treatment methods for fungal balls.^14^, ^15^

In this case, percutaneous surgery was performed because fungal ball was large and transurethral surgery and basket catheter could not adequately remove the fungal ball. Advantage of percutaneous surgery was appropriate for this case. Before the surgery, we were unsure whether the mass was a fungal ball, and antifungal drugs were not used. We regret that we initiated treatment without anticipating mycosis in advance and that we did not use antifungal drugs. Although no established measures have been found to prevent recurrence of fungal ball, some reports suggest continued antifungal therapy is advisable for high‐risk cases.^16^

Complex urinary tract fungal infections are rare, but these infections are often difficult to diagnose and treat and are associated with systemic dissemination which induces a high mortality rate.^4^ It is often difficult to diagnose and choose a treatment method, so it is important to be aware of various treatment methods. More importantly, if the stent is replaced regularly, and the risk of fungal infection is high, medical treatment should be conducted with fungal infections in mind.

## Author contributions

Yoshinori Nakano: Conceptualization; writing – original draft; writing – review and editing. Yohei Sekino: Conceptualization; writing – original draft; writing – review and editing. Kenshiro Takemoto: Writing – review and editing. Takeshi Ueno: Writing – review and editing. Shogo Fujii: Writing – review and editing. Hiroyuki Kitano: Writing – review and editing. Keisuke Hieda: Writing – review and editing. Shinya Ohara: Writing – review and editing. Nobuyuki Hinata: Writing – review and editing.

## Conflict of interest

The authors declare no conflict of interest.

## Approval of the research protocol by an Institutional Review Board

Not applicable.

## Informed consent

Consent to participate and for publication were obtained from the patient.

## Registry and the Registration No. of the study/trial

Not applicable.

## Data Availability

The authors confirm that the data supporting the findings of this study are available in the article.
